# A Wearable Upper Limb Exoskeleton System and Intelligent Control Strategy

**DOI:** 10.3390/biomimetics9030129

**Published:** 2024-02-21

**Authors:** Qiang Wang, Chunjie Chen, Xinxing Mu, Haibin Wang, Zhuo Wang, Sheng Xu, Weilun Guo, Xinyu Wu, Weimin Li

**Affiliations:** 1 Shandong Zhongke Advanced Technology Co., Ltd., Jinan 250100, China; qiang.wang@sdiat.ac.cn (Q.W.);; 2 Guangdong Provincial Key Lab of Robotics and Intelligent System, Shenzhen Institute of Advanced Technology, Chinese Academy of Sciences, Shenzhen 518055, China

**Keywords:** wearable robotics, upper limb assistance, exoskeleton

## Abstract

Heavy lifting operations frequently lead to upper limb muscle fatigue and injury. In order to reduce muscle fatigue, auxiliary force for upper limbs can be provided. This paper presents the development and evaluation of a wearable upper limb exoskeleton (ULE) robot system. A flexible cable transmits auxiliary torque and is connected to the upper limb by bypassing the shoulder. Based on the K-nearest neighbors (KNN) algorithm and integrated fuzzy PID control strategy, the ULE identifies the handling posture and provides accurate active auxiliary force automatically. Overall, it has the quality of being light and easy to wear. In unassisted mode, the wearer’s upper limbs minimally affect the range of movement. The KNN algorithm uses multi-dimensional motion information collected by the sensor, and the test accuracy is 94.59%. Brachioradialis muscle (BM), triceps brachii (TB), and biceps brachii (BB) electromyogram (EMG) signals were evaluated by 5 kg, 10 kg, and 15 kg weight conditions for five subjects, respectively, during lifting, holding, and squatting. Compared with the ULE without assistance and with assistance, the average peak values of EMG signals of BM, TB, and BB were reduced by 19–30% during the whole handling process, which verified that the developed ULE could provide practical assistance under different load conditions.

## 1. Introduction

A wearable power-assisted exoskeleton can assist a wearer in life or work and enhance physical strength. It is mainly used in rehabilitation, military, industrial, and other fields [1,2,3]. It has become a current research hotspot and has made significant progress. The wearable power-assisted exoskeleton assists different parts of the wearer, and the design structure varies greatly. The different types can generally be divided into ULE, lower limb exoskeleton, and whole body exoskeleton. Many researchers have proved they can effectively assist the wearer when developing different exoskeletons [4,5,6,7,8,9]. As a vital component, ULEs show great potential in future applications. A ULE can be worn on the body to assist the upper limbs and ensure wearability [10,11,12]. At the same time, it has the characteristics of flexibility and lightness to prevent the exoskeleton from hindering the normal movement of the human body. Wearing exoskeletons can effectively reduce the energy consumption of upper limbs during operation, improve operation efficiency, and reduce the risk of musculoskeletal injury of wearers. ULEs have been gradually tested in military and industrial fields, and some practitioners have begun to benefit from exoskeleton technology.

In carrying heavy objects, ULEs provide auxiliary force to the wearer’s upper limbs. The exoskeleton comprises rigid structural parts that can effectively transmit power. The biological joints of the human shoulder have seven degrees of freedom. The rigid exoskeleton is mainly designed with fewer degrees of freedom, which cannot meet the degrees of freedom of human biological joints and limits the range of human motion. Moreover, the rotation axis of the exoskeleton cannot be completely aligned with the rotation axis of the human biological joint, which has the risk of causing musculoskeletal injury. The flexible exoskeleton uses flexible components to realize power transmission, which is better in line with human motion and has higher flexibility, avoiding the alignment of the key axis of the exoskeleton with the axis of the human biological joint [13,14]. Park et al. [15] used memory alloy material to weave elastic ribbons and designed wearable ULEs. The temperature change caused the deformation of the memory alloy material, causing the contraction and stretching of the ribbon and the realization of the power of the elbow joint. It can be used for porters or construction workers. The volume is as small as the size of the palm, which is light and flexible. However, using memory alloy materials dramatically affects the ambient temperature, and the temperature control conditions are stringent. José et al. [16] proposed a wearable textile soft exoskeleton that assists shoulder and elbow movement and transmits power using cables. The knitted exoskeleton has a high degree of flexibility. It does not interfere with the normal movement of the wearer. However, the anchor connection and friction in the knitted form affect the efficiency of the exoskeleton in providing auxiliary force. It is designed to provide auxiliary force to the shoulder and elbow. Pont et al. [17] designed an exosuit named ExoFlex. ExoFlex used fabric design combined with specially designed fixing blocks and used Bowden cable to transmit torque. A position super-twisting sliding mode controller was designed and implemented, demonstrating ExoFlex’s adaptability to different wearers’ arms and its stability in user torque. Stretchable fabric, made of elastic rubber and polyamide, is designed as a flexible exoskeleton [18], which aligns more with ergonomics. Through the pre-programmed mode, it assists the arm joint movement and improves durability and flexibility. It is made of non-stretchable nylon tape to resist fabric deformation caused by cable tension. Based on the surface-driven flexible exoskeleton [19], a twisted-column actuator is used to compensate for the muscle activity of the human wearer and change the load when a single-arm or two-arm task is required. It can work together with biological muscle contraction and provide auxiliary force. In an HMI-based exosuit [20], an advanced controller is constructed to fuse EMG signals and human kinematics information, evaluate joint torque, and provide auxiliary force in time. The control method of fusion EMG signals is efficient and accurate, subject to cost and environmental requirements [21], but it is difficult to popularize and apply.

In the case of upper limb assistance, the researchers designed various ways to assist the movement and developed a power-assisted exoskeleton. The rigid power-assisted device is superior to the flexible ULE in efficiency but increases the metabolic cost and distal load, and the flexible exoskeleton has more advantages in transmission. In this paper, a wearable and flexible ULE robot is proposed. Flexible rope is used as the power transmission element to reduce the weight of the rigid structure. The end of the flexible rope is directly connected to the upper limb to reduce the end load and reduce the influence on the flexibility of the upper limb. Based on the KNN algorithm to realize the recognition of human posture, the fuzzy PID control algorithm is used to provide accurate auxiliary force for the wearer. Three muscle groups are tested under 5 kg, 10 kg, and 15 kg loads to analyze the myoelectric activity and evaluate the performance of the ULE auxiliary force.

## 2. Upper Limb Exoskeleton

### 2.1. Design Concept

The main upper limb supporting force is provided by the waist and upper limbs of the human body when weight is lifted from the ground. The upper limbs also provide favorable lifting force. In order to maintain the stability of the center of gravity of the human body, the upper arm is bent in the process of lifting to reduce the risk of body injury. Therefore, providing auxiliary force to the upper limbs can improve the protection of the human body during the handling process, which is a method with great potential [22,23].

The weight that cannot be ignored is the weight added by the wearer after wearing the upper limb assist exoskeleton. The increased weight of the wearer causes an increase in energy consumption after wearing the exoskeleton. However, it has little effect on the body’s energy consumption, as the waist weight is increased by less than 4 kg [24]. Energy consumption is far more than the increased weight of the waist. Since the increased weight of other joints of the upper arm is far away from the center of gravity of the human body, the increased weight of the body will be avoided as much as possible. The upper limb exoskeleton has an extensive range of activities, making the movement more complicated. Therefore, the designed upper limb exoskeleton structure has as few restrictions on upper limb activity as possible.

### 2.2. Mechanical Structure

**Figure 1:**
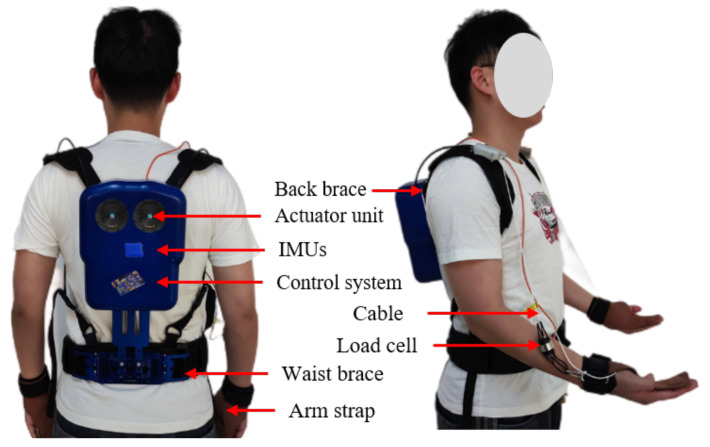
Overall structure of ULE.

Based on the II-A conceptual design, we propose a wearable, flexible upper limb-powered exoskeleton robot that assists the upper limb in a handling scenario. Figure 1 shows the overall layout structure of the ULE. The system consists of control, actuating, and transmission parts, including a tension sensor and an inertial measurement unit (IMU) module. The control and actuating parts are attached to the waist through the connecting rod, which is supported by the waist and bears most of the weight of the system. The transmission part is connected to the upper limb through the flexible cable around the upper side of the shoulder.

**Figure 2:**
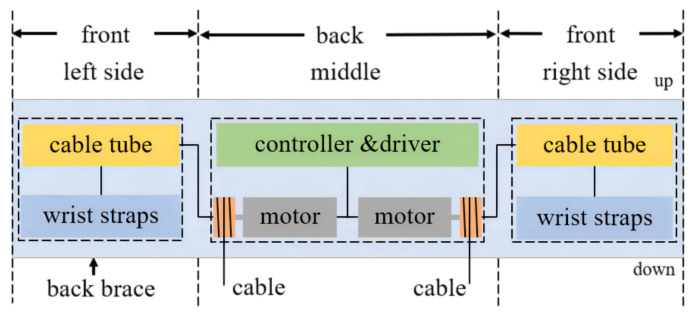
Schematic layout of the transmission units, control units, and actuator units attached to the back brace of the ULE. The execution and control units are directly connected to the back brace. For each upper limb, the transmission unit is connected to the upper limb wrist around the shoulder separately.

**Figure 3:**
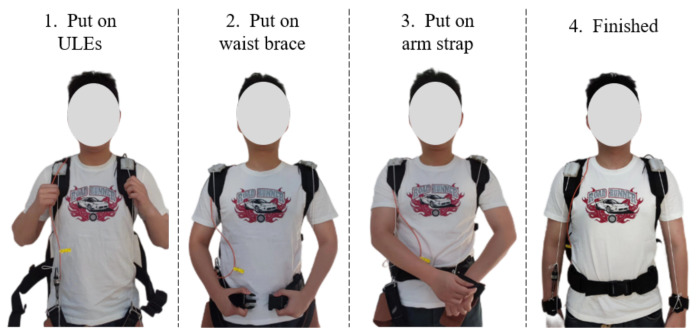
Procedure of putting on the ULE.

Figure 2 shows the layout diagram of the control, actuator, and transmission parts. The system is attached to the human body in the form of waist binding, shoulder binding, and chest binding, as shown in Figure 3. Flexible cables can minimize the weight attached to the upper limb and reduce the upper limb movement restrictions of the ULE on the wearer. The length of the exoskeleton is adjustable, from 42 cm to 45 cm, it is 20 cm wide and can adapt to a height of about 165 cm to 187 cm, and the size radius of the rope winding drum is 15 mm. A lithium battery powers the overall prototype. It can power a DC 24 V. DC motor (T-MOTOR AK80-6) with a maximum peak torque of 12 N.M, and the motor drives the rope to provide auxiliary force.

IMUs (LPMS-B2, Alubi Inc., Guangzhou, China) are installed on the back of the human body and transmit data through Bluetooth. The total mass of the system is 3.36 kg, including the battery. As shown in Figure 3, the ULE is simple and easy to wear, and the wearer can wear it independently and efficiently.

**Figure 4:**
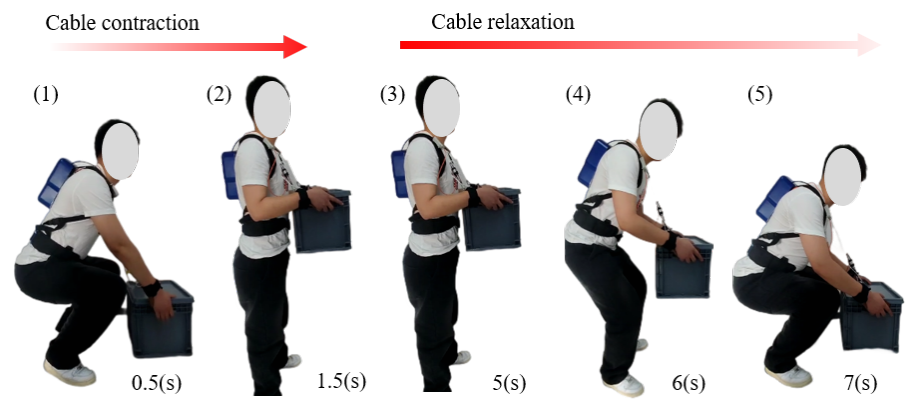
Order of lifting and putting down a heavy object.

The ULE is programmed to collect pose data when the wearer begins to lift heavy objects. Figure 4 shows the process of carrying and holding actions. In the lifting stage, the motor drives the drum to recover the flexible cable, and the flexible transmission tension acts on the heavy object to assist the upper limb lifting.

### 2.3. Control Strategy

**Figure 5:**
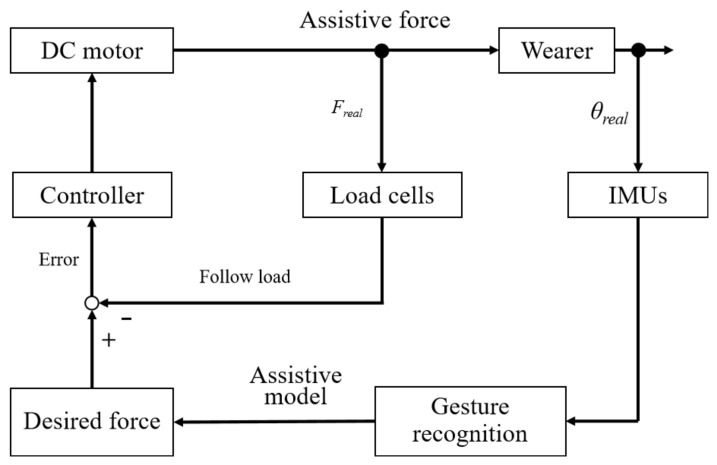
Block diagram of the control strategy. θ real: measured angle by IUMs; $F_{real}$: measured force by load cell.

Figure 5 shows the block diagram of the control strategy. The back motion angle based on the integral of the angular velocity measured by IMUs is obtained. The interpolation method compensates for the missing data of the motion angle, and a 0-degree angle offset compensates for the angular drift. Different assist modes are matched by measuring and comparing the difference between two consecutive angles. The tension feedback control scheme based on a fuzzy PID controller [25] can obtain a smooth response [26,27] for lifting the required tension. A two-in-three-out fuzzy controller is designed. The deviation value e and the deviation change rate E of desired force and the input force are fuzzified, respectively. The output is mapped to the given domain, and the domain of the fuzzy subset is obtained by equalizing the interval. The triangular function is used as the membership function to determine the membership value. Fuzzy rules obtain the size of the output. Based on the weighted average method, the output fuzzy set is solved to obtain the exact value, which is applied to the PID control algorithm parameter adjustment. The control scheme uses a 32-bit microcontroller (stm32f103c8t6, ARM Cortex-M) to achieve the corresponding control functions.

The KNN classification algorithm is one of the most popular and commonly used methods. The advantage is that it can be used in small data sets and for nonlinear classification [28]. The low training time complexity is widely used in machine vision, pedestrian detection, pose recognition, and other fields. This paper uses IMUs to collect data, identify and classify these after preprocessing, map the classification results to different control modes, and cooperate with different actions in the handling process.

Based on the KNN algorithm, the Euclidean distance $d(X_{i},X_{j})$ between the two feature vectors in the sample set is calculated:

(1) $$d\left(X_{i},X_{j}\right)=∥X_{i}-X_{j}∥$$

where $X_{i}$ represents the feature vector of sample *i*, and *X* denotes the sample set: $X=X_{1},\ldots ,X_{N}$, $y_{i}$ is the corresponding category, $y=y_{1},\ldots ,y_{n}$ *F* represents a category, *n* is the number of categories:

(2) $$F=\left\{F_{1},F_{2},\ldots ,F_{N}\right\},y\in F,y\in F$$

For a test sample $X_{m}$, identify the sample most closely related to $X_{m}$ in the known sample set *X*. Assuming the nearest sample is $X_{c}$, and $y_{i}$ = $F_{i}$, then the class discriminant function is as follows:

(3) $$g_{i}\left(X\right)=\min_{X_{c}=P_{i}}d\left(X_{m},X_{c}\right),i=1,2,\ldots ,n$$

Identify the first *K*-known samples nearest to the test sample $X_{m}$. If $K_{i}\in F_{i}$, then the discriminant function for the $F_{i}$ class is:

(4) $$g_{i}\left(X\right)=K_{i},i=1,2,\ldots ,n$$

According to decision rules, the categories of the first *K*-nearest neighbor samples are:

(5) $$\;g_{K}\left(X\right)=\min_{i=1,2,\ldots ,n\;}g_{i}\left(X\right)$$

**Figure 6:**
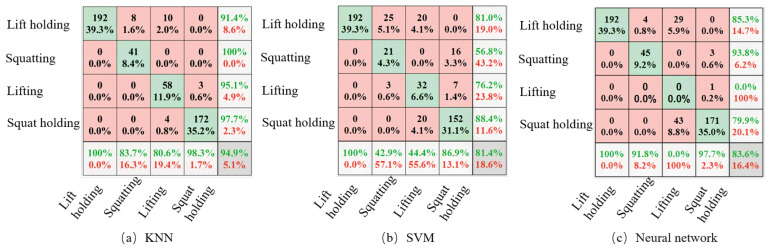
Confusion Matrix. The rows in the above diagram correspond to the prediction class of the data; the correct classification value is on the diagonal; each cell shows the value of the corresponding class and the proportion of the total value; the accuracy value is displayed in the rightmost column described in the above figure; the cell in the lower right corner of the drawing shows the overall accuracy.

In predicting new sample points, the eigenvalues of these points are compared with the corresponding features of the sample points in known categories. The categories with the most similar data to the new sample are used for prediction. The predicted results correspond to four motion states, with each state linked to different control parameters for achieving power assistance in various modes. The four motion states are lifting movement, falling movement, lifting keeping and squatting keeping. IMUs are placed on the back of the human body and kept parallel to the back to detect multi-dimensional data when the human body is lifting, maintaining, and descending. The acquisition frequency of the sensor is set to 2 Hz, and the original data are filtered to obtain a smooth signal. Seventy percent of the data set is taken as the training set and thirty percent as the verification set. Comparing the KNN, support vector machines (SVM) algorithm, and the three-layer neural network algorithm, the accuracy rates are 94.9%, 81.4%, and 83.6%, respectively. KNN shows a higher accuracy rate. The confusion matrix is shown in the following Figure 6.

The rows in the above diagram correspond to the prediction class of the data, and the columns correspond to the test class. The correct classification value is on the diagonal. The value that needs to be correctly classified is on the non-diagonal line. Each cell shows the value of the corresponding class and the proportion of the total value.The accuracy value is displayed in the rightmost column described in the above figure. The recall rate and false negative rate are the percentage of each class of correctly and incorrectly classified instances to all instances, shown in the bottom row. The cell in the lower right corner of the drawing shows the overall accuracy.

Higher predictive and recall values in the above model can accurately identify the corresponding action with more minor errors. In the four action modes, the accuracy of basic lifting and squatting is relatively low, there is some slight confusion between the movements, and the accuracy of squat holding and lift holding is the highest. Nevertheless, the overall accuracy of the model is acceptable for exoskeleton robots.

## 3. Experimental Evaluation

### 3.1. Evaluation of the Range of Motion

The flexible transmission mode shows good adaptability, aiming at the human upper limb’s complex movement, effectively improving the power without interfering with the wearer’s normal working movement.

If the exoskeleton motion range is consistent with the human upper limb motion range, it can provide comfortable wearability to the wearer and reduce the interference of upper limb motion. Previous studies [29,30] evaluated the effect of wearing exoskeletons on the normal range of motion of the human body, which includes the finger part. However, in this study, the study of the finger is removed because the wearable exoskeleton proposed in this paper does not involve the assistance of the finger.

The wearer repeats five handling movements before and after wearing, mainly related to the arm and waist, and uses the three-dimensional motion capture system to achieve motion testing, analysis, and comparison.The upper limbs of the subjects were attached to 10 sampling points. The optical sensor collected upper limb and lumbar motion data at a sampling frequency of 180 Hz. When sampling while wearing the exoskeleton, the same equipment and settings were used for data collection. At this time, the exoskeleton is not in a powered state.

Table 1 shows the comparison results of the wearer in the two states. Similar results of the wearer’s front and back arm movements can be obtained. The main reason for the test results is that the flexible rotation method does not interfere with the wearer’s upper limb movement. These results show that the flexible transmission mode is light and dexterous and has little interference with the upper limb movement. In shoulder horizontal extension and flexion, the limit is relatively large compared to other movement parts. The main reason for the decrease from 89.6° to 83.2° is a slight motion interference between the flexible ropes fixed on the shoulder. Nevertheless, the impact is acceptable in the actual handling work.

### 3.2. Evaluation of Assistive Force

The auxiliary performance of the proposed exoskeleton is based on five healthy adults, consisting of three males and two females, wearing the proposed ULE and performing all the handling actions. All wearers wear the same exoskeleton because the ULE can be adjusted to fit the body shape and shape of five different wearers. The participant information is shown in Table 2.

In this experiment, the wearer activates the ULE by pressing the power switch to provide assistive power when they need it. The muscle activity data of the wearer in the case of wearing the exoskeleton are compared with those in the case of not wearing the ULE, and the assist performance of the ULE is analyzed.

The experiment was approved by the Medical Ethics Committee of Shenzhen Institute of Advanced Technology ((SIAT)-IRB-221215-H0632), and the content of the experiment and its possible impact were explained to all the participants before experimenting. None of the subjects reported any history of medical illness. The subjects are guaranteed to be in healthy physical condition.

During this evaluation, selecting the electromyography (EMG) muscle position most conducive to reflecting the upper limb movement and connecting the electrodes is necessary [31,32,33]. The surface EMG sensor was attached to the muscle of the wearer’s right arm. Based on the process of carrying heavy objects, the three muscles are highly correlated, the EMG signal intensity is significant, and the acquisition quality is high. The EMG signals of three muscles were collected in the brachioradialis muscle (BM), triceps brachii (TB), and biceps brachii (BB).

Each wearer is tested according to the handling process, including lifting, holding, and squatting. The lifting action refers to lifting the weight after bending and squatting. The EMG information of the arm was collected from the low position to the high position. In contrast, the holding action refers to lifting the weight at a certain height and holding it for 5 s. The EMG information of the arm-related muscle groups was collected under the state of continuous force. The other movements of the arm were prohibited during the experiment; the squatting action refers to placing the weight on the ground after maintaining the action, collecting the arm EMG signal in the process. The EMG signal was collected during handling under the three weight levels of 5 kg, 10 kg, and 15 kg. After each level test was completed, the wearer was fully rested, and the independence of each test’s data was maintained as much as possible.

According to the standard steps of EMG signal acquisition before the test, EMG signal inspection during data acquisition, band-pass filtering, and rectification is performed using a fourth-order zero-lag Butterworth filter of 20–450 Hz. Eliminate interference from other devices or external environments and filter 20–150 Hz data. Next, the data is low-pass filtered to create a linear envelope. The 400 ms moving window was used to determine the average EMG amplitude in the MVC, and the EMG amplitude was normalized when the highest average was used for evaluation. The wearer’s EMG signals were collected under 5 kg, 10 kg, and 15 kg, respectively, in the lifting, holding, and squatting actions, and the data were normalized. The values after processing are shown in the following figure.

**Figure 7:**
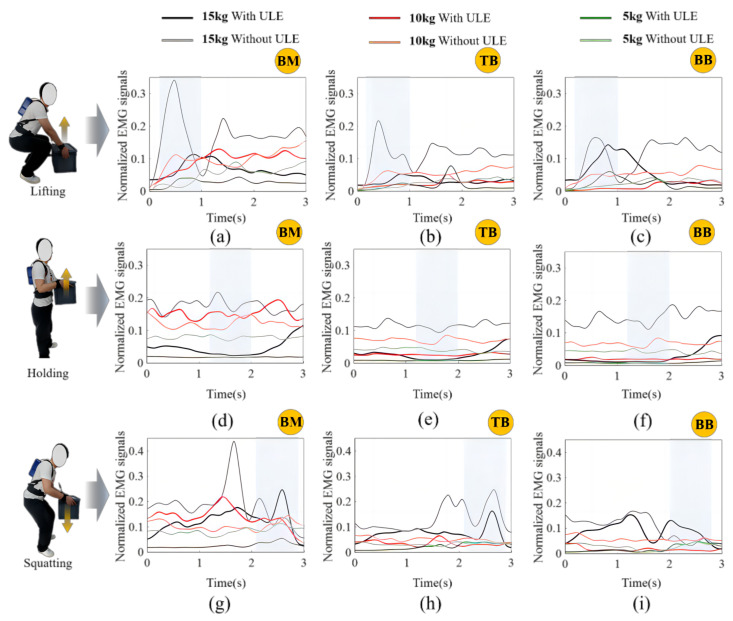
Normalized EMG signals. Normalized EMG linear envelopes of right arm muscle (BM, TB, BB) during the lifting, holding, and squatting actions under 5 kg, 10 kg, and 15 kg conditions, respectively, showing a comparison diagram with assistance and without assistance, where black indicates the ULE under 15 kg, with assistance. Light black indicates the ULE under 15 kg, without assistance; red indicates the ULE of 10 kg with assistance; light red indicates the ULE of 10 kg, without assistance; green indicates the ULE under 5 kg conditions with assistance; and light green indicates the ULE under 5 kg without assistance, where (**a**–**c**) represents the normalized EMG signal amplitude of BM, TB, BB muscle groups in the lifting state, and the rectangle shadow section represents the main assist phase. (**d**–**i**) is a similar display.

As shown in Figure 7, the normalized EMG linear envelope diagram represents BM, TB, and BB muscle activity in the three stages of the handling process under different weights of 5 kg, 10 kg, and 15 kg. In summary, muscle activity decreased under the condition of ULE assistance compared with that under the condition of without ULE assistance. However, BM, TB, and BB muscles showed relatively strong muscle activity in the non-assisted mode during the lifting and squatting stages. The possible reason is the lag of sensor data collection. During the assist delay of the ULE, in the rising and squatting stages, the muscle compensates for the lack of assist or the existence of flexible line friction, and the auxiliary force transmitted to the user is reduced, which ultimately leads to insufficient auxiliary force, and BM, TB, BB muscle activity increases. This fact is positive; the ULE can effectively reduce fatigue, as is proved, but also provides a further basis for friction. Table 3, Table 4 and Table 5 show the assist efficiency of the ULE under regular work. Under different weights of 5 kg, 10 kg, and 15 kg, the assist efficiency of BM, TB, and BB muscle activities is shown in the three stages of the handling process.

The handling efficiency is based on the ratio of the average value of the normalized EMG signal under the condition of ULE and without ULE. The Table 3, Table 4 and Table 5 show the handling efficiency of lifting, holding, and squatting stages under 5 kg, 10 kg, and 15 kg load conditions. All handling efficiency is between 9% and 38%. Table 6 data show the average efficiency of the entire handling process. Under 5 kg, 10 kg, and 15 kg load conditions, the average work efficiency of BM, TB, and BB is between 19% and 38%. This data shows that the ULE reduces muscle activity during handling and reduces the risk of physical loss during the wearer’s long-term continuous operation.

**Figure 8:**
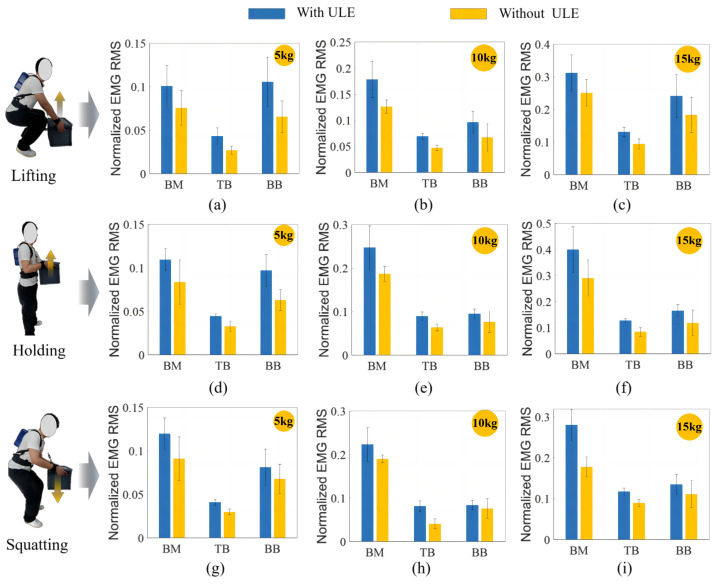
RMS of normalized EMG. Bars and error bars represent the average values and standard deviations of RMS, which are calculated based on the normalized EMG RMS of multiple different stages. (**a**–**c**) represents the mean and standard deviation of arm muscle (BM, TB, BB) EMG with and without ULE at 5 kg, 10 kg, and 15 kg conditions, respectively. (**d**–**f**) and (**g**–**i**) are similar displays. Adjacent histograms are statistically significant (p<0.05).

RMS is one of the key indicators used to evaluate muscle fatigue [34,35]. The RMS of the normalized EMG in the three stages is shown in Figure 8. The figure shows that the value without ULE assistance is still the highest, and the EMG signals are reduced when wearing ULE, indicating that the ULE provides practical assistance. All adjacent pairs of histograms were also subjected to a *t*-test, which revealed that all comparisons were statistically significant (*p* < 0.05).

## 4. Conclusions

This work demonstrates a lightweight and flexible ULE robot auxiliary device for providing auxiliary force when handling heavy objects, which can reduce muscle activity during the wearer’s handling. Based on the KNN algorithm, automatic recognition of action posture is realized during the wearer’s handling process. The flexible cable transmits torque to provide auxiliary force to the wearer. The system allows the wearer to reduce the interference of upper limb arm activity as much as possible in the non-assisted mode. Five participants conducted experiments based on the EMG acquisition test of TM, TB, BB, and muscle groups under 5 kg, 10 kg, and 15 kg load conditions. The data showed that the average muscle activity of the subjects’ TM, TB, and BB decreased, compared with the ULE and without ULE, by between 19% and 30%, and the power performance of the exoskeleton was verified.

Despite this, optimization and improvement are needed. In terms of design, the system structure is lightweight to minimize the energy consumption of the ULE. Further, upper limb movement is complex and lacks periodic action. The proposed ULE can provide auxiliary force during handling, and the best assist mode can be explored for different scenarios. In addition, evaluating the ULE system should be systematic and multifaceted [36,37,38]. When carrying out handling movements, the use of advanced sensors can be deployed to measure the impact on other biological joints [39,40]. Comprehensive muscle evaluation is conducive to the exploration of assist strategies. Based on this, our ultimate goal is to develop an efficient and accurate assist system to improve the wearer’s work efficiency and reduce physical damage.

## Figures and Tables

**Table 1:** Range of Motion.

Movement	Range of Motion (degree)	
	Without ULE	With ULE
Shoulder adduction and abduction	170.3	168.2
Shoulder extension and flexion	207.3	206.2
Shoulder horizontal extension and flexion	89.6	83.2
Elbow extension and flexion	128.5	126.6
Forearm supination and pronation	150.1	149.6

**Table 2:** Participant Information.

Subjects	Age (years)	Height (cm)	Weight (kg)
1	24	184	85
2	27	165	60
3	31	180	80
4	23	166	51
5	33	179	76

**Table 3:** Assist Efficiency of Lifting.

Condition	BM	TB	BB
5 kg	25%	37%	38%
10 kg	29%	32%	30%
15 kg	19%	28%	24%

**Table 4:** Assist Efficiency of Holding.

Condition	BM	TB	BB
5 kg	24%	26%	35%
10 kg	24%	28%	19%
15 kg	27%	34%	28%

**Table 5:** Assist Efficiency of Squatting.

Condition	BM	TM	BB
5 kg	24%	27%	16%
10 kg	15%	23%	9%
15 kg	36%	23%	17%

**Table 6:** Mean Assist Efficiency of Handling Process.

Condition	BM	TB	BB
5 kg	24%	30%	29%
10 kg	23%	27%	19%
15 kg	27%	28%	23%

## Data Availability

The data that support the findings of this study are available on request from the corresponding author.

## Abbreviations

The following abbreviations are used in this manuscript:

ULE	Upper Limb Exoskeleton
KNN	K-Nearest Neighbors
BM	Brachioradialis muscle
TB	triceps brachii
BB	biceps brachii
EMG	electromyography
